# Asbestos Exposure and Development of Pulmonary Pleomorphic Carcinoma in a Non-smoker: A Rare Non-small Cell Lung Cancer

**DOI:** 10.7759/cureus.37860

**Published:** 2023-04-20

**Authors:** Andrew L Alejo, Riya A Patel, Krutarth K Pandya, Krishna Bodrya, Lawrence Goldstein, Lori Hemrock

**Affiliations:** 1 College of Medicine, Northeast Ohio Medical University, Rootstown, USA; 2 Internal Medicine, Trumbull Regional Medical Center, Warren, USA

**Keywords:** risk factor, non-small cell lung cancer, malignancy, lung cancer, asbestosis, pulmonary pleomorphic carcinoma

## Abstract

Pulmonary pleomorphic carcinoma (PPC) is a subtype of non-small cell lung cancer that is extremely rare and carries a poor prognosis due to its inadequate response to treatment. Patients that present with PPC often exhibit similar symptoms of other malignancies of the lung, making it hard for clinicians to distinguish between each type. However, cytology and gene mutation testing are two approaches that can aid physicians in an accurate and definitive diagnosis.

We present a case of an 88-year-old male patient with a diagnosis of pulmonary pleomorphic carcinoma after experiencing recurrent sanguineous pleural effusions. The patient had no smoking history but did have a history of asbestos exposure and pulmonary fibrosis. The patient underwent thoracotomy with pleurodesis and analysis of the surgical pleural biopsy specimen stained positive for markers indicative of PPC. The pathology report was also consistent with the associated cell morphology.

Lung cancer is the leading cause of mortality due to cancer in the United States, and exposure to certain substances contributes to the development of these poorly treatable lung malignancies. Smoking and asbestos exposure are well known to act synergistically with each other as risk factors in developing these lung malignancies. In addition to clinical suspicion, screening for these risk factors with laboratory values and imaging is important to diagnose these rare cases of lung malignancies.

## Introduction

Pulmonary pleomorphic carcinoma (PPC) is a rare subset of non-small cell lung cancer (NSCLC) that is estimated to be seen in approximately 0.1-0.4% of all primary lung malignancies [1]. According to the World Health Organization (WHO) classification, PPC is defined as a lung malignancy with pleomorphic, sarcomatous, or sarcomatoid elements. In particular, it is a poorly differentiated adenocarcinoma, squamous cell carcinoma, or large cell carcinoma with sarcomatoid components of the spindle or giant cells that are at least 10% of the cells [2].

PPC has a poor prognosis due to its inadequate response to systemic chemotherapy and radiation, with an estimated five-year survival rate of 12% [3]. There have been limited case reports that have shown successful treatment by resection or immune checkpoint inhibitors; however, they have low success rates [4,5]. In this study, we report a PPC case in a patient that had recurrent bloody pleural effusions and a history of asbestos exposure with pulmonary fibrosis. The tumor was located in the same anatomical region where the recurrent pleural effusions occurred and based on pathological and cytological findings, the patient was diagnosed with PPC.

## Case presentation

Our patient is an 88-year-old Caucasian male with a past medical history of pulmonary fibrosis, atrial fibrillation, and chronic kidney disease who presented to the emergency department with complaints of chest pain for one week. The pain was substernal with no radiation to his neck, jaw, or back. He became progressively short of breath and dyspneic with exertion with an occasional, dry, nonproductive cough. He denied chills, rigors, nausea, vomiting, headache, or diaphoresis. The patient was started on amoxicillin P.O. earlier in the week due to suspected pneumonia. He had no smoking history but did reveal he was exposed to asbestos for over 10 years during his occupation.

**Figure 1:**
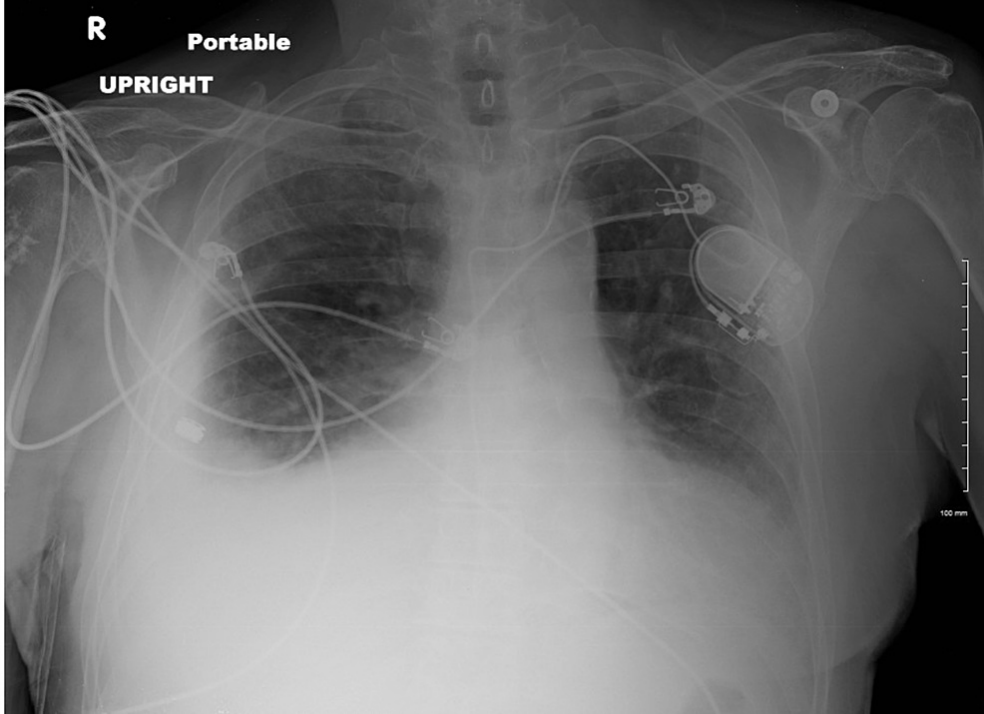
Chest X-ray (1st Admission)

**Figure 2:**
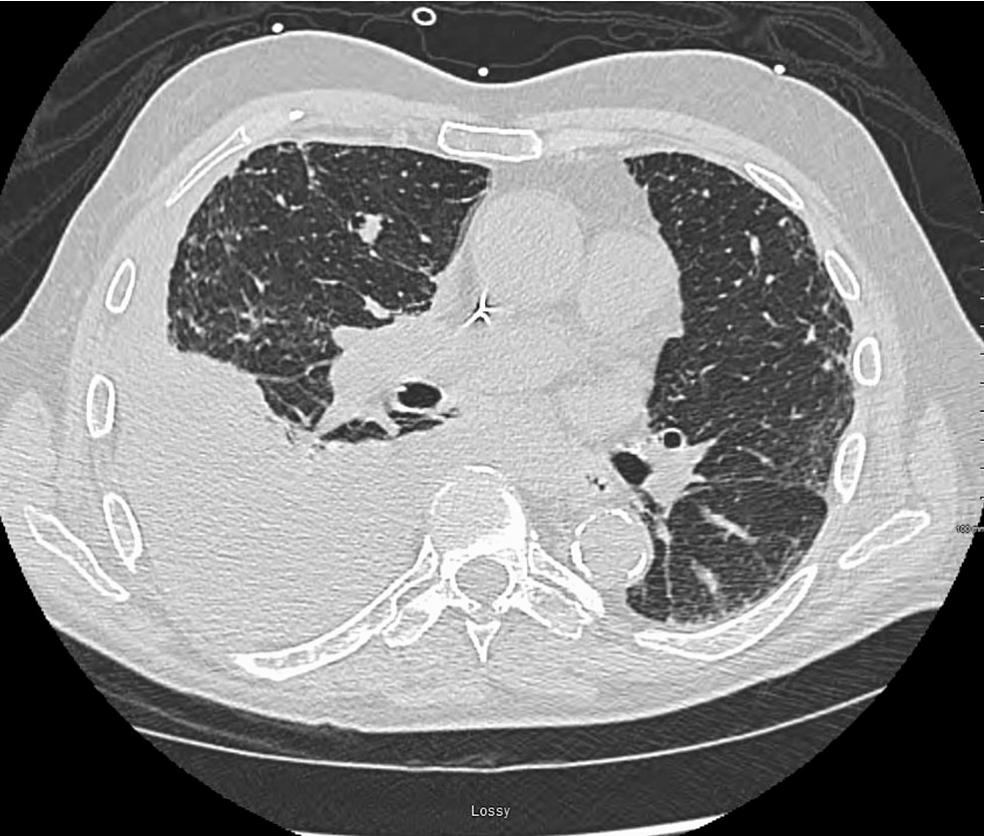
Chest CT (1st Admission)

Workup for chest pain was initiated, which included a complete blood count, basic metabolic panel, chest X-ray, and electrocardiogram, with significant findings including an elevated WBC, 77.8% neutrophils, blood urea nitrogen (BUN), creatinine, atrial fibrillation with a rapid ventricular rate, with a right bundle-branch block and a small-to-moderate pleural effusion with right basilar airspace disease (Table 1, Figure 1). The patient underwent thoracentesis with the removal of 1500 cc of serosanguinous fluid. The decision to proceed with the placement of an indwelling pleural drainage catheter was made due to the recurrence of the pleural effusion days later from which 1100 cc of grossly bloody fluid was obtained (Figure 2). Cytology report from pleural fluid was negative for malignancy but did show reactive atypia of the mesothelium. The patient was discharged to home with home health services and was advised to follow up with his primary care physician.

**Table 1:** Laboratory Values

	Hospital Admission 1 ED	Hospital Admission 1 Discharge	Hospital Admission 2 ED	Reference
White Blood Cells	15.4x10^3^	18.1x10^3^	22.6x10^3^	3.5-10.5x10^3^/uL
Red Blood Cells	3.26x10^6^	2.45x10^6^	2.43x10^6^	4.32-5.72x10^6^/uL
Hemoglobin	10.1	7.3	7.4	13.5-17.5 gm/dL
Hematocrit	30.7	23.9	23.3	38.8-50%
Neutrophils %	77.8	77.6	87	34-71%
Blood Urea Nitrogen	29	32	32	7-18mg/dL
Creatinine	1.43	0.86	1.07	0.7 to 1.3 mg/dL
Glucose	116	98	104	70 to 99 mg/dL

**Figure 3:**
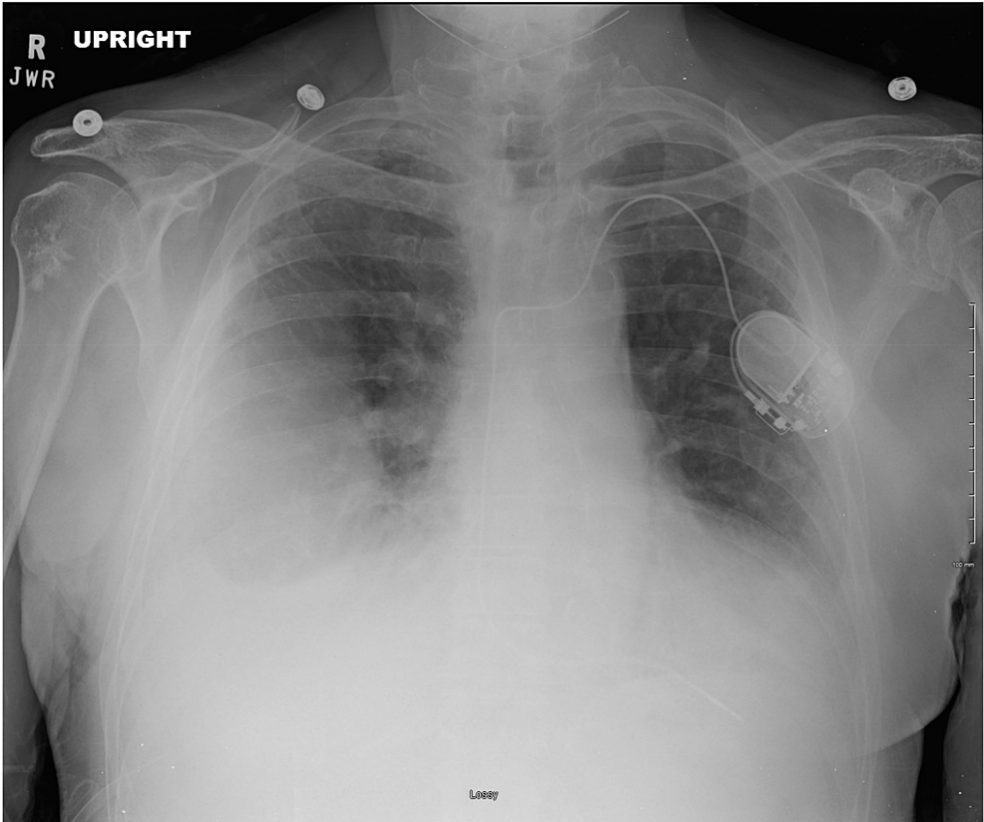
Chest X-ray (2nd Admission)

**Figure 4:**
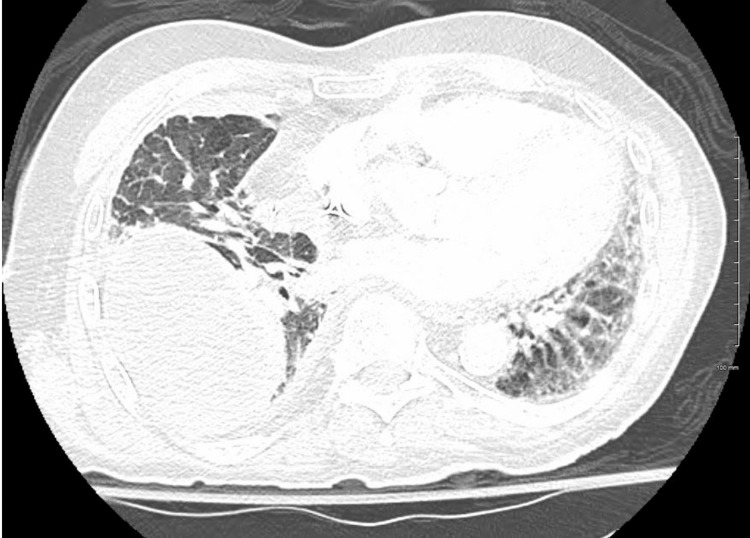
Chest CT (2nd Admission)

The patient returned to the emergency department one week later with his pleural catheter draining bright red blood. Chest X-ray showed worsening of pleural effusion (Figure 3). The patient only complained of extreme fatigue and denied chest pain, dyspnea, abdominal pain, nausea, and vomiting. Labs indicated an elevated WBC with neutrophilia and low hemoglobin (Table 1). A computed tomography scan of the chest showed a lower lobe infiltrate along with a loculated pleural effusion and IV piperacillin-tazobactam was started (Figure 4). With recurrent loculated bloody pleural effusions, a thoracic surgery consult was requested again to obtain a good-quality surgical specimen. The patient went for an open thoracotomy surgery, which went well without any complications, and the patient was transferred to the intensive care unit because of his increased oxygen requirement of 8 liters via nasal cannula. He subsequently developed acute hypoxic respiratory failure and required non-invasive ventilation. With worsening respiratory failure, the patient and family requested no intubation or mechanical ventilation for the patient.

Five days later, the patient was still dependent on a non-invasive ventilator while the pathological surgical specimen of the pleura showed pleomorphic carcinoma of the lung with negative markers for mesothelioma (Table 2), and an oncology consult was requested. With worsening respiratory failure and a guarded prognosis, after an extensive discussion with the patient and his family, they elected comfort care measures. The patient expired soon after the withdrawal of care and making him comfortable with appropriate medications.

**Table 2:** Cytology Report

Marker	Expression
WT1	Negative
Mesothelin	Negative
PDL-1	Strongly Positive
TTF-1	Positive
Pancytokeratin	Positive
Calretinin	Focally and weakly positive
CK5/6	Negative
CK7	Negative
Napsin a	Negative
P 40	Negative
PSA	Negative
Gata 3	Negative
CDX2	Negative
CK20	Negative
D2-40	Negative

## Discussion

Pulmonary pleomorphic carcinoma is a rare class of NSCLC that has been scarcely reported in the literature and accounts for only 0.1-0.4% of all primary lung cancers [1]. Patients with this malignancy experience common clinical symptoms such as hemoptysis, chest pain, dyspnea, fever, and cough. Additionally, the demographic of the patients reported includes a mean age of 60 to 65 years old, a male-to-female ratio of 2:1, and a history of smoking (60-90%) [3,6,7]. The exact incidence could not be reported in different ethnic groups due to the rarity. PPC is known to have an aggressive clinical course, with worse clinical outcomes compared to other NSCLCs [3].

Patients that have a previous smoking history have been shown to be associated with developing this rare cancer. Smoking is associated with cancers developing in the upper lobes since these areas of the lung are most frequently in contact with the carcinogens in the smoke. It has been reported that in PPC, the most common site of growth is the right upper lobe [3,8]; however, in our patient, cancer and recurrent pleural effusions were located in the right lower lobe. Additionally, he presented with recurrent sanguineous pleural effusions of the same anatomical location, which should always raise clinical suspicion for malignancy.

The patient had an unusual presentation with previous exposure to asbestos and no smoking history. Only one other case report has shown that a patient developed PPC with asbestos exposure [9]. As it is known that smoking and asbestos exposure interact synergistically for the causation of lung cancer, it is important to note that asbestos exposure alone can lead to the development of PPC as shown in our patient. His significant length of exposure to asbestos is in line with the temporal pattern of exposure and development of lung malignancy [10]. His past medical history of pulmonary fibrosis additionally may have predisposed him to this condition as well.

Current medical treatment for this rare lung cancer is limited but there has been some evidence of promising results with certain checkpoint inhibitors in combination with radiation therapy [1,5,11]. In addition to the tumor markers, testing for mutations for EGFR, KRAS, and ALK can be helpful in certain patients, which can potentially help in deciding the treatment plan [12]. PPC can frequently express PDL-1 like our patient did, which can help decide treatment pathways as well [13]. Surgical resection can be attempted if the patient is a good surgical candidate without metastases. Pulmonary pleomorphic carcinoma should be considered a differential diagnosis when NSCLC is suspected.

## Conclusions

We report on a rare pleomorphic carcinoma that was discovered after the patient presented with recurrent bloody pleural effusions in the same anatomical location of the lung. Asbestosis may play a role in the development of PPC. As lung cancer is the leading cause of mortality due to cancer in the United States, exposure to certain substances contributes to the development of these poorly differentiated lung malignancies. Although PPC is associated with a poor prognosis, a high index of suspicion and early identification with possible surgical resection may improve the outcomes for this patient population.
